# Machine Learning Approach for Prediction of the Test Results of Gonadotropin-Releasing Hormone Stimulation: Model Building and Implementation

**DOI:** 10.3390/diagnostics13091550

**Published:** 2023-04-26

**Authors:** Yu-Shao Chen, Chung-Feng Liu, Mei-I Sung, Shio-Jean Lin, Wen-Hui Tsai

**Affiliations:** 1 Department of Pediatrics, Chi Mei Medical Center, No. 901 Zhonghua Rd., Yongkang District, Tainan City 710402, Taiwan; 2 Medical Research Department, Chi Mei Medical Center, No. 901 Zhonghua Rd., Yongkang District, Tainan City 710402, Taiwan; 3 Graduate Institute of Medical Sciences, College of Health Sciences, Chang Jung Christian University, No. 1 Changda Rd., Gueiren District, Tainan City 711301, Taiwan

**Keywords:** central precocious puberty, diagnosis, gonadotropin-releasing hormone stimulation test, machine learning

## Abstract

Precocious puberty in girls is defined as the onset of pubertal changes before 8 years of age, and gonadotropin-releasing hormone (GnRH) agonist treatment is available for central precocious puberty (CPP). The gold standard for diagnosing CPP is the GnRH stimulation test. However, the GnRH stimulation test is time-consuming, costly, and requires repeated blood sampling. We aimed to develop an artificial intelligence (AI) prediction model to assist pediatric endocrinologists in decision making regarding the optimal timing to perform the GnRH stimulation test. We reviewed the medical charts of 161 girls who received the GnRH stimulation test from 1 August 2010 to 31 August 2021, and we selected 15 clinically relevant features for machine learning modeling. We chose the models with the highest area under the receiver operating characteristic curve (AUC) to integrate into our computerized physician order entry (CPOE) system. The AUC values for the CPP diagnosis prediction model (LH ≥ 5 IU/L) were 0.884 with logistic regression, 0.912 with random forest, 0.942 with LightGBM, and 0.942 with XGBoost. For the Taiwan National Health Insurance treatment coverage prediction model (LH ≥ 10 IU/L), the AUC values were 0.909, 0.941, 0.934, and 0.881, respectively. In conclusion, our AI predictive system can assist pediatric endocrinologists when they are deciding whether a girl with suspected CPP should receive a GnRH stimulation test. With proper use, this prediction model may possibly avoid unnecessary invasive blood sampling for GnRH stimulation tests.

## 1. Introduction

Precocious puberty in girls is traditionally defined as the onset of pubertal changes before 8 years of age. If left untreated, it can lead to compromised final adult height and early menarche [1]. Furthermore, negative emotional and behavioral consequences have been reported, such as substance abuse, peer pressure, self-image concerns, social isolation, early sexual behavior, conduct issues, social isolation, truancy, and having multiple sexual partners [1,2]. Precocious puberty can be classified into two types: central (gonadotropin-dependent) and peripheral (gonadotropin-independent). Central precocious puberty (CPP) results from earlier maturation and activation of the hypothalamic–pituitary–gonadal axis. It is usually idiopathic in girls, though it can also be caused by pathological conditions, such as central nervous system (CNS) tumors, CNS injury, or genetic syndromes (neurofibromatosis type 1, tuberous sclerosis, Sturge–Weber Syndrome, etc.). Peripheral precocious puberty is gonadotropin-independent. It results from endogenous or exogenous sources of sex steroids, such as congenital adrenal hyperplasia, McCune–Albright syndrome, gonadal/adrenal tumors, or exogenous sex steroid exposure [1].

The increasing prevalence of obesity has been associated with earlier onset of thelarche in girls [3]. Moreover, researchers of a cross-sectional study of 20,654 apparently healthy urban Chinese girls showed that up to 19.57% of these girls had evidence of breast development at 8 years of age [4]. However, these girls did not necessarily have activation of the hypothalamic–pituitary–gonadal axis and may have had premature thelarche only. Therefore, identifying girls with central precocious puberty from those who do not is important. The gold standard for diagnosing CPP is an evaluation of the hypothalamic–pituitary–gonadal axis maturation through the gonadotropin-releasing hormone (GnRH) stimulation test [3,5]. After GnRH injection, blood sampling is performed three to five times at different time points to measure serum gonadotropin concentration changes. The diagnosis of CPP is traditionally made if the peak serum concentration after stimulation is ≥5 IU/L [6].

Since the mid-1980s, long-acting GnRH agonists have been used for the treatment of CPP. Continuous GnRH agonist stimulation on pituitary gonadotrophs causes desensitization and decreased LH release [7]. In Taiwan, the criteria for reimbursement from the National Health Insurance (NHI) regarding long-acting GnRH agonist treatment in CPP include a peak LH ≥ 10 IU/L after a GnRH stimulation test. If a girl obtains a peak LH level ≥5 IU/L but <10 IU/L, she likely needs to repeat the GnRH stimulation test a few months later in order to apply for the reimbursement of GnRH agonist treatment to determine if the criterion of LH ≥ 10 IU/L is met.

The GnRH stimulation test is time-consuming, costly, and requires repeated blood sampling, which is uncomfortable for patients [3]. The optimal timing for arranging the GnRH stimulation test in a girl with suspected CPP is a challenge frequently faced by pediatric endocrinologists. If a girl has a high chance of showing a negative result, she is less likely to benefit from the GnRH stimulation test. However, if the test is arranged too late into the clinical course, treatment for the patient may be delayed. The local insurance policy is another consideration. Taking Taiwan as an example, obtaining the probability of receiving a stimulated peak LH ≥ 10 IU/L would be very helpful for pediatric endocrinologists. In this study, we aimed to develop an artificial intelligence (AI) prediction model for the results of the GnRH stimulation test, which may help pediatric endocrinologists make decisions regarding the optimal timing for arranging the GnRH stimulation test.

## 2. Methods

### 2.1. Study Design, Setting, and Samples

We retrospectively reviewed the medical charts of all pediatric female patients who received the GnRH stimulation test in Chi Mei Medical Center between 1 August 2010 and 31 August 2021. We obtained approval from the Institutional Review Board of the hospital before data collection (IRB No.: 11011-001).

**Figure 1:**
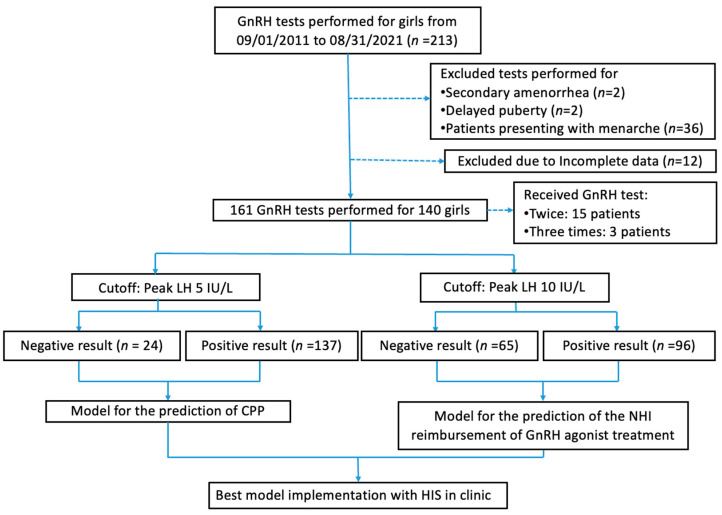
Study Flow. CPP, central precocious puberty; GnRH, gonadotropin-releasing hormone; HIS, hospital information system; LH, luteinizing hormone; NHI: National Health Insurance.

We excluded patients with menarche because a very high chance of a positive GnRH stimulation test result can be assumed in these cases without assistance from a prediction model. We also excluded patients whose medical records had missing items. Two girls received the GnRH stimulation test due to secondary amenorrhea, and two other girls received it due to delayed puberty. We excluded these four girls from our study because their GnRH stimulation tests did not indicate suspected CPP. The overall study flow is described in detail in Figure 1.

### 2.2. Features and Outcome Variables

We collected data from electronic medical records of the following variables generally available for data collection in clinical practice: chronological age (CA), age of thelarche, height, height SDs, weight, weight SDs, BMI, paternal height, maternal height, mid-parental height (MPH), predicted adult height (PAH), annual growth rate, bone age (BA), Tanner stage (for breast and pubic hair development), lab result (random serum LH, FSH, E2), and birth history (gestational age, birth body weight). We calculated MPH via the following formula: (paternal height + maternal height − 13)/2. We calculated the z-scores for height and weight according to the data published by Chen et al. in 2010 [8]. We interpreted the BA using the Greulich and Pyle method [9]. PAH was calculated using BA and published data (Chen et al., 2010) based on Taiwanese children and adolescents [8]. The GnRH stimulation tests were performed with an intravenous bolus of 0.1 mg gonadorelin acetate. Blood samples were obtained for the baseline and at 30, 60, and 90 min after gonadorelin injection. Serum LH, FSH, and E2 were measured using the Abbott Architect i2000SR immunoassay analyzer (Abbott Laboratories, Irving, TX, USA). We chose two cutoff values of stimulated LH as the prediction outcomes for establishing our AI models: LH ≥ 5 IU/L (level for CPP diagnosis) and ≥10 IU/L (level for NHI reimbursement for CPP treatment in Taiwan). We compared the demographics of the stimulation test results of the positive group (LH ≥ cutoff after stimulation) to that of the negative group (LH < cutoff after stimulation).

### 2.3. Statistical Analysis and Model Building

For practical implementation, we selected features based on statistical significance and clinical experts’ opinion. We then randomly stratified the data into a training dataset (70% data) for model building and a testing dataset (30% data) for model validation. Because the number of negative test result cases was lower than that of positive test result cases, we used the SMOTE method (synthetic minority over-sampling technique) to improve the data imbalance in the training dataset [10]. We paired each outcome with 4 machine learning algorithms to build the predictive models. These algorithms were as follows: (1) logistic regression (LR), (2) random forest (RF), (3) LightGBM, and (4) XGBoost. We used Python and scikit-learn machine learning tools. We used a grid search with 5-fold cross validation to tune the hyper-parameters to build the best models based on the training dataset.

### 2.4. Model Evaluation and Practical Implementation

After building the models, we used the test dataset to validate the models according to well-defined model performance indicators: accuracy, sensitivity, specificity, and AUC (area under the receiver operating characteristic curve). We regarded the model with the highest AUC value as the best model and used it to further implement a prediction system for practical use. We built the model with the Python language and scikit-learn libraries. We developed a web-based prediction system with Microsoft Visual Studio^®^ 2017 and integrated it into the existing hospital information system (HIS). The prediction system can immediately capture feature values from the HIS and display the risk probabilities for predicting LH > 5 and LH > 10.

## 3. Results

### 3.1. Enrollment and Baseline Statistical Tests

As presented in Figure 1, during the study period of 1 September 2011 to 31 August 2021, 161 GnRH stimulation tests were performed for 140 pediatric female patients with early thelarche and suspected CPP. A total of 15 Patients received the GnRH test twice due to negative results from the first test. A total of three patients received the GnRH test three times due to negative results from the first two tests. Using the peak LH cutoff level of 5 IU/L, we allocated 24 tests to the “Peak LH < 5 IU/L group” and 137 tests to the “Peak LH ≥ 5 IU/L group”. For the LH cutoff level of 10 IU/L, we allocated 65 tests to the “Peak LH < 10 IU/L group” and 96 tests to the “Peak LH ≥ 10 IU/L group”. The detailed baseline characteristics and grouping based on the LH cutoff values of 5 and 10 are shown in Table 1 and Table 2.

### 3.2. Characteristics and Features

When choosing the cutoff level of ≥5 IU/L for stimulated peak LH, there was a borderline difference in BMI classification between the negative test result and the positive test result groups (*p* = 0.046). The positive test result group (stimulated peak LH ≥ 5 IU/L) had a higher frequency of underweight BMIs (0.73% vs. 0%), normal BMIs (73.72% vs. 62.50%), and overweight BMIs (16.79% vs. 8.33%), and we observed a lower frequency for obesity (8.76% vs. 29.17%) when compared with the negative test result group. The positive test result group also had a significantly higher annual growth rate (8.35 vs. 7.1 cm/yr, *p* = 0.007). In addition, the positive test result group also had a significantly lower frequency of Tanner stage B2 (27.74% vs. 70.83%) and a higher frequency of Tanner stage B3 (51.09% vs. 25%), Tanner stage B4 (19.71% vs. 4.17%), and Tanner stage B5 (1.46% vs. 0%). For the laboratory characteristics, the positive test result group had a significantly higher baseline FSH (3.66 vs. 2.19, IU/L, *p* < 0.001), baseline LH (1.42 vs. 0.21, IU/L, *p* < 0.001), and LH/FSH ratio (0.34 vs. 0.09, *p* < 0.001).

We noted similar findings when choosing the cutoff level of ≥10 IU/L for stimulated peak LH. The positive test result group (stimulated peak LH ≥ 10 IU/L) had a significantly lower frequency of Tanner stage B2 (23.96% vs. 49.23%) and a higher frequency of Tanner stage B3 (50% vs. 43.08%), Tanner stage B4 (23.96% vs. 7.69%), and Tanner stage B5 (2.08% vs. 0%) when compared with the negative test result group. The positive test result group also had a significantly higher annual growth rate (8.8 vs. 7.22, cm/yr, *p* < 0.001). For the laboratory characteristics, the positive test result group had a significantly higher random FSH (3.87 vs. 2.8, IU/L, *p* < 0.001), random LH (1.83 vs. 0.37, IU/L, *p* < 0.001), and LH/FSH ratio (0.43 vs. 0.12, *p* < 0.001). In the positive test result group, we observed a significantly greater height (135.44 vs. 133.88cm, *p* < 0.04). However, Ht SDs had no significant difference. Bone age was significantly more advanced in the positive test result group (10.71 vs. 10.47, yr, *p* = 0.047). However, we found no difference in BA advancement (BA-CA) or in ΔBA/ΔCA between the two bone age films.

### 3.3. Model Building and Evaluation

**Figure 2:**
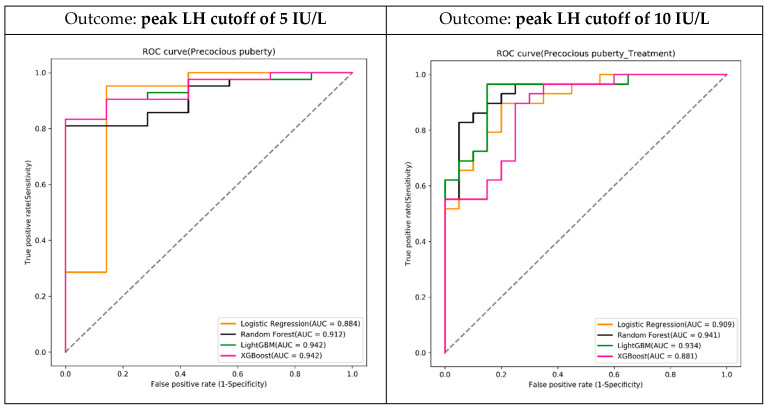
Receiver operating characteristic curves (ROCs) of test results. AUC, area under the ROC curve; LH, luteinizing hormone.

We used four machine learning algorithms, LR, RF, LightGBM, and XGBoost, to build the prediction models with the training dataset. We used a grid search with five-fold cross-validation to tune the hyper-parameters for each algorithm to obtain the best model. We tested the prediction models with the test dataset and evaluated the metrics of their accuracy, sensitivity, specificity, and AUC. Among the four algorithms, the XGBoost algorithm had the best performance with the highest AUC (see Table 3, Figure 2).

We further compared our machine learning algorithms to a practical scoring system based on breast Tanner stage, basal LH, and basal FSH, which was proposed in a recent study from Taiwan [11] (see Table 4). The AUC for all machine learning algorithms was higher than the scoring system. Accuracy and specificity were also better when adjusting to the same sensitivity.

### 3.4. Prediction System Development and User Evaluation

**Figure 3:**
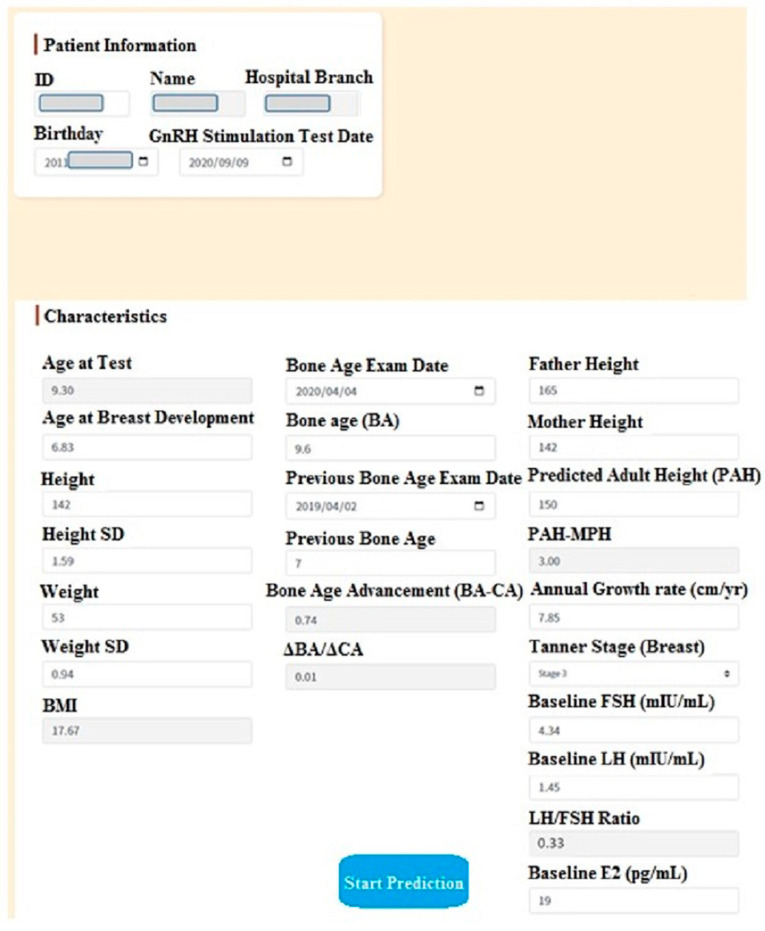
Screenshot (translated into English) of the interactive AI prediction system (interface page to capture/fill in necessary feature values). AI, artificial intelligence; BA, bone age; BMI, body mass index; CA, chronological age; E2, estradiol; FSH, follicle-stimulating hormone; GnRH, gonadotropin-releasing hormone; ID, identity; LH, luteinizing hormone; MPH, mid-parental height; PAH, predicted adult height; SD, standard deviation.

The LightGBM prediction model for the LH cutoff value of 5 IU/L was the best model because it had the highest AUC and accuracy, and the random forest prediction model was the best model for the LH cutoff value of 10 IU/L. We therefore used these best models for the development and deployment of a clinical prediction system. The AI Center and Department of Information Systems of Chi Mei Medical Center embedded the best models in a web-based AI system for predicting the test results of gonadotropin-releasing hormone stimulation tests (see Figure 3). It launched in March 2022.

We showed the AI system to three clinicians in the pediatric endocrinology department and gained high recognition. The system is a potentially useful tool in the decision-making process of whether the GnRH stimulation test should be performed. The graphic display of AI-calculated probability can also aid clinicians with explanations to patients and parents.

### 3.5. Case Scenarios

**Figure 4:**
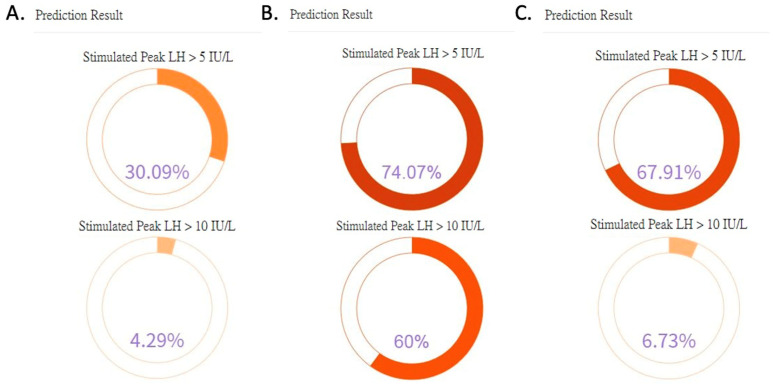
(**A**–**C**) Results page (translated into English) of the AI prediction system showing predicted results for three cases. LH, luteinizing hormone.

As shown in Figure 4A, a physician performed an AI prediction for a patient in the outpatient clinic. The result showed a low probability for both stimulated LH > 5 IU/L (30.09%) and stimulated LH > 10 IU/L (4.29%). With such results, the clinician may need to re-evaluate the patient and reconsider arranging the GnRH stimulation test. In Figure 4B, another patient showed a high probability for both stimulated LH > 5 IU/L (74.07%) and stimulated LH > 10 IU/L (60%). The result was consistent with the clinician’s evaluation, and the patient would likely benefit from arranging the GnRH stimulation test. As shown in Figure 4C, the prediction results for the third patient showed a high probability for stimulated LH > 5 IU/L (67.91%). However, the prediction result for stimulated LH > 10 IU/L was low (6.73%). This prediction result suggests that the patient is likely to have ongoing central precocious puberty but may not yet fulfill the cutoff for the NHI reimbursement of GnRH analog treatment. The patient would likely benefit from a close follow-up and the arrangement of a GnRH stimulation test after a short period of time. Although the AI prediction system cannot replace the importance of the clinician’s clinical evaluation, the above three scenarios show that the AI prediction system can be used to check for consistency with the clinical evaluation. When the prediction result is inconsistent with the clinical evaluation, a reminder is presented to the clinician to reassess the patient and consider the option of further close follow-up rather than immediate arrangement of the GnRH stimulation test.

## 4. Discussion

In this study, we established an AI prediction system and integrated it into our computerized physician order entry (CPOE) system to predict GnRH stimulation test results. We demonstrated that, if the GnRH stimulation test is still clinically needed, an AI prediction system is an ideal method to save time, reduce costs, and avoid unnecessary repeated blood samplings.

Many studies have attempted to simplify or substitute the GnRH stimulation test. Various studies have suggested various cutoff values for basal LH, with variable sensitivity [11,12,13,14,15,16]. Other studies have tried to reduce the number of blood samplings required for the GnRH stimulation test, suggesting that a single blood sampling at 30 min or 40 min post GnRH injection may be adequate [11,17,18]. The subcutaneous administration of GnRH was also proposed, but multiple blood samplings are still required under such a protocol [19]. Despite all these attempts, the GnRH stimulation test is still considered the gold standard test and is more frequently used.

Aside from trying to simplify or replace the GnRH test, using scoring systems or AI models to predict the probability of a positive test result may provide another solution to this problem. Researchers of a recent study from Taiwan proposed a practical scoring system using breast Tanner stage, basal LH, and basal FSH [11]. The scoring system had 76% sensitivity and 72% specificity. The stimulated LH cutoff level was set as 10 IU/L in the study due to the NHI reimbursement criteria for GnRH analog treatment. Researchers of another study from Shanghai proposed a CPP risk score model, which classifies patients into low-, median-, and high-risk groups [20]. They used a stimulated peak LH ≥ 5 IU/L and a peak LH/FSH ratio ≥ 0.6 for the diagnosis of CPP. Similar to our study, researchers of a study from Guangzhou proposed prediction models using machine learning algorithms [21]. However, the diagnostic criteria include either peak LH levels ≥ 10 IU/L or peak LH levels ≥ 5 IU/L combined with a peak LH/FSH ratio ≥ 0.6.

In this study, the features we selected were routinely evaluated for suspected CPP patients in clinical practice. Via history taking, the onset age of thelarche and the age of the patients during the visit were routinely recorded. Further inquiry about the height of parents can help calculate the mid-parental height (MPH). Via physical examination, height, weight, BMI, annual growth rate, and Tanner staging were obtained. Because patients presented at different ages, we used height SDs and weight SDs in our models to better represent the heights and weights of the patients compared with girls of the same age. Laboratory workup, including bone age, LH, FSH, and LH/FSH ratio, can also be easily obtained at the outpatient clinic.

Compared with the non-CPP group (stimulated peak LH < 5 IU/L), the CPP group (stimulated peak LH ≥ 5 IU/L) had a significantly higher frequency of underweight, normal, and overweight BMIs, and the frequency of obesity was lower. This can be explained by the fact that obese girls presenting breast lumps do not necessarily have activation of the hypothalamic–pituitary–gonadal axis [3]. However, these girls may be considered candidates for the GnRH stimulation test because of the earlier onset of thelarche. The results of the GnRH stimulation tests for these individuals would likely be negative. Therefore, the clinician should be careful not to overestimate breast development in obese girls [7]. On the other hand, obese children may have blunted LH response during the GnRH stimulation test as a result of LH suppression due to androgen/estrogen excess [22].

The Tanner stage is commonly used as the standard for breast and pubic hair development ratings in clinical practice [23]. In our study, the Tanner staging for breast development had a significantly more advanced distribution in the positive test result group, regardless of the stimulated peak LH cutoff level chosen. However, we found no significant difference in Tanner staging for pubic hair development and the presence of axillary hair. This can be explained by the finding that breast bud appearance is the first pubertal sign in girls. However, the appearance of pubic hair can occur before, after, or together with puberty onset [24]. Adrenal-derived androgens can cause the appearance of pubic hair and axillary hair. Premature adrenarche may present with an advanced bone age but without breast development [25]. The hypothalamic–pituitary–gonadal axis is not activated in such circumstances.

A higher growth velocity and advanced bone age were shown as factors for predicting positive results in the GnRH stimulation test, with a rapid growth velocity suggested as the most useful predictive factor [26]. In our study, annual growth rate was significantly higher in the positive test result group, regardless of the stimulated peak LH cutoff level chosen. In addition to bone age, we also included factors such as BA-CA and ΔBA/ΔCA. However, we found no significant difference in these factors between the positive and negative test result groups. Intraoperator and interoperator variability in bone age determination using an atlas-based method is not uncommon [27]. This provides an explanation for our results because bone age reports are generated by different radiologists in our hospital.

In the laboratory features, our results show a higher basal FSH, basal LH, and basal LH/FSH ratio in the positive GnRH stimulation test result group. This is consistent with previous studies, in which basal LH, FSH, and LH/FSH ratio were found to be significantly higher in individuals with a positive GnRH stimulation test result [11,26].

The strength of the prediction models in our study is that our models can be used to predict the probability of either stimulated LH ≥ 5 IU/L or stimulated LH ≥ 10 IU/L. A physician may have different considerations while arranging the GnRH stimulation test. The prediction model for CPP diagnosis (stimulated LH ≥ 5 IU/L) can help decide whether the GnRH stimulation test is helpful in confirming the diagnosis for a girl with suspected CPP. However, if the reimbursement of the GnRH agonist treatment is also a consideration, the prediction model for treatment coverage (stimulated LH ≥ 10 IU/L) can be used together with the prediction model for CPP diagnosis (stimulated LH ≥ 5 IU/L). When used together, the two models can help predict whether the test result would reveal that the patient does not have CPP (LH < 5 IU/L), that the patient has CPP but does not meet NHI treatment criteria (≥5 IU/L but <10 IU/L), or that the patient has CPP and meets the NHI treatment criteria (LH ≥ 10 IU/L).

However, our model still has some limitations. First, this study has a retrospective design. Potential problems, such as missing or inaccurate data, could have occurred. However, all the features used in our model are basic information obtained via history taking and physical exams. The lab data, including LH, FSH, and E2, were also routinely obtained in girls who received the GnRH stimulation test. Therefore, we did not encounter any missing data during data collection. Second, we interpreted bone age reports from different radiologists, and interoperator variability could have occurred, even when using the same Greulich and Pyle method. We did not include pelvic echo information for the same reason because such measurements can be even more operator-dependent. Third, we collected our data from a single medical center in Southern Taiwan. The study population is mostly composed of members of the Han Chinese ethnic group, with few ethnically Southeastern Asian individuals. Therefore, our model may not be suitable in other populations or regions. Finally, although our model shows a good AUC, as clinicians, we also need to take sensitivity into account, since we would not want to miss the diagnosis on any patient. To address this, we can lower the classification threshold to improve model sensitivity, but false alarms (false positives) may increase and affect model performance. Therefore, we would suggest using our model to assist predicting the result of the GnRH stimulation test and seek better timing with an appropriate evaluation threshold for test arrangement (0.5 by default), rather than using it to rule out the diagnosis. The diagnosis should be based on the overall clinical picture instead. Furthermore, though the model shows a good AUC, we did not prospectively validate the model. We plan to address this issue in our future work via real-time prediction using the AI system launched in March 2022.

## 5. Conclusions

Our machine learning models can provide valuable information to pediatric endocrinologists when they need to decide whether a girl with suspected CPP should receive a GnRH stimulation test. The two models may be used alone or together for predicting whether the stimulated LH level would be <5 IU/L, between 5 and 10 IU/L, or ≥10 IU/L. With the assistance of these prediction models, pediatric endocrinologists can choose the optimal timing for arranging GnRH stimulation tests. To the best of our knowledge, very few studies have used machine learning approaches to build prediction models regarding the optimal timing for arranging GnRH stimulation tests, and even fewer have implemented real-time AI system prediction in a clinical setting. Thus, the results of our study have profound academic and practical novelty and value. We call for future researchers to consider including more parameters to improve prediction performance. We also encourage broadening the retrospective data to include multiple centers.

## Figures and Tables

**Table 1:** Demographics of groups, with a cutoff value of peak LH levels at 5 IU/L.

Variable	Negative Test Result (Peak LH < 5 IU/L) (*n* = 24)	Positive Test Result (Peak LH ≥ 5 IU/L) (*n* = 137)	*p*-Value
Age at test (yr)	8.55 (0.63)	8.51 (1.17)	0.389
Age of breast development (yr)	6.55 (1.14)	6.50 (1.00)	0.777
Ht (cm)	133.52 (4.53)	135.04 (9.16)	0.120
Ht SD	0.79 (0.69)	1.07 (1.15)	0.137
Wt (kg)	34.74 (8.18)	32.57 (6.98)	0.443
Wt SD	1.08 (1.05)	0.82 (0.95)	0.340
BMI	19.35 (3.67)	17.69 (2.46)	0.057
BMI classification			0.046 *
underweight	0	1 (0.73)	
NR	15 (62.50)	101 (73.72)	
overweight	2 (8.33)	23 (16.79)	
obese	7 (29.17)	12 (8.76)	
Father Ht (cm)	172.79 (6.76)	171.42 (5.94)	0.304
Mother Ht (cm)	157.06 (5.69)	157.47 (4.71)	0.673
Mid-parental height (MPH, cm)	158.43 (5.00)	157.94 (4.15)	0.818
Bone age (yr)	10.36 (1.22)	10.65 (1.46)	0.205
BA advancement (yr)	1.81 (1.10)	2.14 (0.98)	0.297
ΔBA/ΔCA	1.67 (1.46)	1.98 (1.55)	0.337
Predicted adult height (PAH, cm)	154.40 (4.78)	153.89 (4.98)	0.885
PAH-MPH (cm)	−4.02 (5.92)	−4.05 (4.96)	0.911
Annual growth rate (cm/yr)	7.10 (1.55)	8.35 (2.19)	0.007 *
Tanner stage for breast development			0.001 *
2	17 (70.83)	38 (27.74)	
3	6 (25.00)	70 (51.09)	
4	1 (4.17)	27 (19.71)	
5	0	2 (1.46)	
Tanner stage for pubic hair development			0.344
1	23 (95.83)	109 (79.56)	
2	1 (4.17)	19 (13.87)	
3	0	8 (5.84)	
4	0	1 (0.73)	
Axillary hair			0.742
None	22 (91.67)	118 (86.13)	
Present	2 (8.33)	19 (13.87)	
Baseline FSH (mIU/mL)	2.19 (1.00)	3.66 (1.84)	<0.001 *
Baseline LH (mIU/mL)	0.21 (0.20)	1.42 (1.72)	<0.001 *
Baseline E2 (pg/mL)	21.57 (16.46)	26.77 (20.00)	0.146
LH/FSH ratio	0.09 (0.05)	0.34 (0.30)	<0.001 *
GA (wk)	38.23 (1.89)	38.64 (1.31)	0.544
Premature birth			0.213
No	21 (87.50)	129 (94.16)	
Yes	3 (12.50)	8 (5.84)	
BBW (g)	2792.83 (493.94)	2964.34 (368.74)	0.093
Size for gestational age			0.113
SGA	15 (62.50)	110 (80.29)	
AGA	8 (33.33)	23 (16.79)	
LGA	1 (4.17)	4 (2.92)	
LH peak (mIU/mL)	3.75 (1.02)	22.39 (20.09)	<0.001 *

Categorical variables are presented as frequencies with a percentage, *n* (%). Continuous variables are presented as means with standard deviations (mean ± SD). * *p* < 0.05. AGA, appropriate for gestational age; BA, bone age; BBW, birth body weight; BMI, body mass index; CA, chronological age; E2, estradiol; FSH, follicle-stimulating hormone; GA, gestational age; GnRH, gonadotropin-releasing hormone; LGA, large for gestational age; LH, luteinizing hormone; MPH, mid-parental height; PAH, predicted adult height; SD, standard deviation; SGA, small for gestational age.

**Table 2:** Demographics of groups, with a cutoff value of peak LH levels at 10 IU/L.

Variable	Negative Test Results (Peak LH < 10 IU/L) (*n* = 65)	Positive Test Results (Peak LH ≥ 10 IU/L) (*n* = 96)	*p*-Value
Age at test (yr)	8.48 (0.85)	8.54 (1.25)	0.144
Age of breast development (yr)	6.44 (0.95)	6.55 (1.07)	0.216
Ht (cm)	133.88 (6.07)	135.44 (9.99)	0.040 *
Ht SD	0.93 (0.87)	1.10 (1.23)	0.292
Wt (cm)	33.17 (6.87)	32.71 (7.43)	0.996
Wt SD	0.93 (0.99)	0.80 (0.95)	0.420
BMI	18.40 (3.04)	17.62 (2.45)	0.150
BMI classification			0.542
underweight	0	1 (1.04)	
NR	44 (67.69)	72 (75.00)	
overweight	11 (16.92)	14 (14.58)	
obese	10 (15.38)	9 (9.38)	
Father Ht (cm)	172.12 (6.21)	171.29 (5.97)	0.182
Mother Ht (cm)	157.79 (5.31)	157.15 (4.53)	0.562
Mid-parental height (MPH, cm)	158.46 (4.38)	157.72 (4.19)	0.225
Bone age (yr)	10.47 (1.09)	10.71 (1.62)	0.047 *
BA advancement (yr)	1.99 (0.90)	2.16 (1.06)	0.473
ΔBA/ΔCA	1.79 (1.41)	2.04 (1.61)	0.347
Predicted adult height (PAH, cm)	154.10 (5.02)	153.88 (4.90)	0.918
PAH-MPH (cm)	−4.36 (5.17)	−3.83 (5.06)	0.391
Annual growth rate (cm/yr)	7.22 (1.75)	8.80 (2.17)	<0.001 *
Tanner stage for breast development			0.001 *
2	32 (49.23)	23 (23.96)	
3	28 (43.08)	48 (50.00)	
4	5 (7.69)	23 (23.96)	
5	0	2 (2.08)	
Tanner stage for pubic hair development			0.078
1	59 (90.77)	73 (76.04)	
2	5 (7.69)	15 (15.62)	
3	1 (1.54)	7 (7.29)	
4	0	1 (1.04)	
Axillary hair			0.803
None	56 (86.15)	84 (87.50)	
Present	9 (13.85)	12 (12.50)	
Baseline FSH (mIU/mL)	2.80 (1.44)	3.87 (1.91)	<0.001 *
Baseline LH (mIU/mL)	0.37 (0.38)	1.83 (1.89)	<0.001 *
Baseline E2 (pg/mL)	22.83 (15.59)	28.13 (21.65)	0.114
LH/FSH ratio	0.12 (0.07)	0.43 (0.32)	<0.001 *
GA (wk)	38.64 (1.50)	38.53 (1.35)	0.466
Premature birth			1.000
no	61 (93.85)	89 (92.71)	
yes	4 (6.15)	7 (7.29)	
BBW(g)	2935.89 (393.59)	2940.73 (394.54)	0.989
Size for gestational age			0.889
SGA	52 (80.00)	73 (76.04)	
AGA	11 (16.92)	20 (20.83)	
LGA	2 (3.08)	3 (3.12)	
LH peak (mIU/mL)	6.07 (2.23)	28.78 (20.95)	<0.001 *

Categorical variables are presented as frequencies with a percentage, *n* (%). Continuous variables are presented as means with standard deviations (mean ± SD). * *p* < 0.05. AGA, appropriate for gestational age; BA, bone age; BBW, birth body weight; BMI, body mass index; CA, chronological age; E2, estradiol; FSH, follicle-stimulating hormone; GA, gestational age; GnRH, gonadotropin-releasing hormone; LGA, large for gestational age; LH, luteinizing hormone; MPH, mid-parental height; PAH, predicted adult height; SD, standard deviation; SGA, small Therefor gestational age.

**Table 3:** Performance of the two outcome predictive models (peak LH cutoff of 5 IU/L and peak LH cutoff of 10 IU/L).

Predictive Models with Each ML Algorithm	Accuracy	Sensitivity	Specificity	AUC	95% CI (AUC)
Peak LH cutoff of 5 IU/L					
Logistic regression	0.939	0.952	0.857	0.884	0.686–0.999
Random forest	0.857	0.881	0.714	0.912	0.825–0.998
LightGBM	0.898	0.905	0.857	0.942	0.877–0.999
XGBoost	0.878	0.905	0.714	0.942	0.876–0.999
Peak LH cutoff of 10 IU/L					
Logistic regression	0.837	0.862	0.800	0.909	0.830–0.988
Random forest	0.878	0.897	0.850	0.941	0.876–0.999
LightGBM	0.857	0.862	0.850	0.934	0.849–0.999
XGBoost	0.837	0.931	0.700	0.881	0.786–0.976

AUC, area under the receiver operating characteristic curve; LH, luteinizing hormone; ML, machine learning.

**Table 4:** Comparison of machine learning algorithm to another clinical scoring system (Yeh et al. [11]) for predicting LH Peak Cutoff of 10 IU/L.

Algorithms	Accuracy	Sensitivity	Specificity	AUC	AUC 95% CI	*p*-Value
Scoring system	0.735	0.966	0.400	0.683	0.568–0.798	-
Logistic regression	0.796	0.966	0.550	0.909	0.830–0.988	0.001
Random forest	0.878	0.966	0.750	0.941	0.876–0.999	<0.001
LightGBM	0.918	0.966	0.850	0.934	0.849–0.999	<0.001
XGBoost	0.837	0.966	0.650	0.881	0.786–0.976	0.003

Comparing the two AUCs using DeLong’s test. AUC, area under the receiver operating characteristic curve.

## Data Availability

Not applicable.
